# A Novel Rhoptry Protein as Candidate Vaccine against *Eimeria tenella* Infection

**DOI:** 10.3390/vaccines8030452

**Published:** 2020-08-12

**Authors:** Xingju Song, Xu Yang, Taotao Zhang, Jing Liu, Qun Liu

**Affiliations:** 1 National Animal Protozoa Laboratory, College of Veterinary Medicine, China Agricultural University, Beijing 100083, China; b20163050366@cau.edu.cn (X.S.); yangxu129@cau.edu.cn (X.Y.); zhangtaotao2019@tmu.edu.cn (T.Z.); liujingvet@cau.edu.cn (J.L.); 2 Key Laboratory of Animal Epidemiology of the Ministry of Agriculture, College of Veterinary Medicine, China Agricultural University, Beijing 100083, China

**Keywords:** *Eimeria tenella*, sporozoite, rhoptries protein, anticoccidial vaccines

## Abstract

*Eimeria tenella* (*E. tenella*) is a highly pathogenic and prevalent species of *Eimeria* that infects chickens, and it causes a considerable disease burden worldwide. The secreted proteins and surface antigens of *E. tenella* at the sporozoite stage play an essential role in the host–parasite interaction, which involves attachment and invasion, and these interactions are considered vaccine candidates based on the strategy of cutting off the invasion pathway to interrupt infection. We selected two highly expressed surface antigens (SAGs; Et-SAG13 and Et-SAG) and two highly expressed secreted antigens (rhoptry kinases Eten5-A, Et-ROPK-Eten5-A and dense granule 12, Et-GRA12) at the sporozoite stage. Et-ROPK-Eten5-A and Et-GRA12 were two unexplored proteins. Et-ROPK-Eten5-A was an *E. tenella*-specific rhoptry (ROP) protein and distributed in the apical pole of sporozoites and merozoites. Et-GRA12 was scattered in granular form at the sporozoite stage. To evaluate the potential of rEt-ROPK-Eten5-A, rEt-GRA12, rEt-SAG13 and rEt-SAG proteins as a coccidiosis vaccine, the protective efficacy was examined based on survival rate, lesion score, body weight gain, relative body weight gain and oocyst output. The survival rate was significantly improved in rEt-ROPK-Eten5-A (100%) and rEt-GRA12 (100%) immune chickens compared to the challenged control group (40%). The average body weight gains of rEt-ROPK-Eten5-A, rEt-GRA12, rEt-SAG13 and rEt-SAG immunized chickens were significantly higher than those of unimmunized chickens. The mean lesion score and oocyst output of the rEt-ROPK-Eten5-A immunized chickens were significantly reduced compared to unimmunized challenged chickens. These results suggest that the rEt-ROPK-Eten5-A protein effectively triggered protection against *E. tenella* in chickens and provides a useful foundation for future work developing anticoccidial vaccines.

## 1. Introduction

Coccidiosis is caused by the genus *Eimeria*, and it is one of the most widespread and economically detrimental diseases affecting the global poultry industry [1,2]. The annual loss due to coccidiosis exceeds $3 billion USD globally [3]. *Eimeria tenella* (*E. tenella)* is one of the most harmful species due to its wide prevalence and high pathogenicity [4]. *E. tenella* parasitizes chicken cecal epithelial cells and leads to reduced body weight gains, epithelial cell damage and death [5].

Conventional control strategies primarily rely on anticoccidial drugs [3,6]. However, alternative control strategies are urgently needed due to the rapid emergence of drug-resistant parasites, the high cost of new drug development and the increasing legislation restrictions on the use of anticoccidial drugs [7,8]. Although live oocyst vaccines are used commercially in some regions, virulence variation and the high cost of vaccine production restrict the application of live vaccines [9,10]. Only CoxAbic was successfully commercialized. However, CoxAbic is a subunit vaccine prepared from *E. maxima* affinity-purified gametocyte antigens (APGAs), and it is limited by a complicated purification process and high production cost [3,11]. These drawbacks have driven the development of new control strategies, especially the screening of effective immunoprotective proteins for the development of a subunit vaccine [7].

*E. tenella* belongs to the phylum Apicomplexa, and it has rhoptries (ROP), micronemes (MIC) and dense granule (GRA) secretory organelles. These secretory organelles produce a large number of secretory proteins that mediate parasite invasion and survival [12]. Proteomic and genomic sequence profiling of apicomplexans showed that many secreted proteins were ROP proteins [13,14,15]. The secreted proteins and surface antigens (SAGs) play a critical role in host–parasite interactions involving attachment and invasion, which are considered vaccine candidates [9]. The extracellular sporozoite stage of *E. tenella* is critical for invasion, and it is immunologically vulnerable and functionally important for the parasite [16]. Therefore, the screening of surface antigens and secreted antigens that are highly expressed at the sporozoite stage is significant for the development of anticoccidial vaccines. The strategy for these vaccines is to block parasite infection by cutting off the invasion process. Several sporozoite proteins of *Eimeria* were identified as anticoccidial vaccine candidates. A sporozoite antigen (micronemes 1, EtMIC1) improved the efficacy against *E. tenella* [17]. A rhomboid-like protease (ETRHO1) also showed value as a vaccine candidate because it imparted partial protection to chickens against *E. tenella* [18]. The sporozoite-specific SAG1 induced partial protective immunity as a recombinant protein vaccine [19,20,21]. Antigen refractile body protein (EtSO7) is located in sporozoite refractile bodies, and it provoked cellular and humoral immune responses to provide significant protection against cecal coccidiosis in immunized chickens [16]. Immune mapped protein 1 (IMP1) was recently identified as an anticoccidial vaccine candidate, and it is localized on the sporozoite cell membrane [22,23].

The present study (i) characterized four sporozoite antigens from *E. tenella*, (ii) expressed the four sporozoites antigens and located two unexplored proteins (Et-ROPK-Eten5-A and Et-GRA12) in different stages of parasites and (iii) evaluated the protective efficacy of these four antigens to develop an effective vaccine against coccidiosis.

## 2. Materials and Methods

### 2.1. Ethics Statement

All animal experiments were performed in strict accordance with the recommendations of the Guide for the Care and Use of Laboratory Animals of the Ministry of Science and Technology of China. The Institutional Animal Care and Use Committee of China Agricultural University approved all experimental procedures (approval number: AW05(7)069102-2).

### 2.2. Parasites and Animals

*E. tenella* was maintained and propagated in two-week-old coccidia-free SPF chickens, as previously described [24]. The oocysts were collected and purified as previously described [25]. SPF chickens and female BALB/c mice were obtained from Merial Animal Health Co., Ltd. (Beijing, China). Sterilized food and clean water without anti-coccidia drugs were provided in sufficient supply.

### 2.3. Bioinformatic Analysis

The nucleotide sequences and amino acid sequences were downloaded from ToxoDB (https://toxodb.org/toxo/). The amino acid sequence alignment was performed using Basic Local Alignment Search Tool in NCBI (https://blast.ncbi.nlm.nih.gov/Blast.cgi) and Clustal X software version 1.83 (http://www.clustal.org/). A phylogenetic tree was constructed using the neighbor-joining method in MEGA software (version 5.05, State College, PA, USA) (http://www.megasoftware.net/). The signal peptide was analyzed using the SignalP 4.0 server (http://www.cbs.dtu.dk/services/SignalP/). Transcriptional level data were obtained from ToxoDB uploads from Walker et al. [26]. The kinase domain was predicted using SMART (http://smart.embl-heidelberg.de/).

### 2.4. Cloning, Expression and Purification of Four Sporozoite Antigens

Total RNA was isolated using Trizol^®^ (Life Technologies, Carlsbad, MD, USA), and cDNA was synthesized using TransScript One-Step gDNA Removal and cDNA Synthesis SuperMix (Transgen Biotech, Beijing, China). The full coding sequence of Et-ROPK-Eten5-A without the signal peptide sequence was amplified from sporozoite cDNA using the primers in Supplementary Table S1 (Supplementary Materials). High-fidelity enzyme (ransgen Biotech, Beijing, China) was used for the PCR reaction, and the PCR protocol was used as recommended in the instructions. The expression plasmid was pET28a (+) (Novagen, Darmstadt, Germany), which was preserved in the Key Laboratory of Animal Parasitology (Beijing, China). The pET28a (+) plasmid was linearized using PCR and ligated with the Et-ROPK-Eten5-A sequence via seamless cloning (Vazyme Biotech, Co., Ltd., Nanjing, China). The recombinant plasmid was transformed into *Transetta* (DE3) cells (TransGen, Beijing, China). The expression and purification procedures for recombinant Et-ROPK-Eten5-A protein (rEt-ROPK-Eten5-A) were performed following the manufacturer’s protocol (Novagen, Darmstadt, Germany). Briefly, the cells were induced for 5 h at 37 °C with 1 mM isopropyl-D-1-thiogalactopyranoside (IPTG), harvested and broken via ultrasonication. The recombinant protein was purified using a Ni^2+^ affinity column. Protein concentration was measured using micro-BCA protein assay reagent (Pierce, Rockford, IL, USA). The amplification, expression and purification procedures of rEt-GRA12, rEt-SAG13 and rEt-SAG were the same as those for rEt-ROPK-Eten5-A.

### 2.5. Sera

Negative serum was obtained from coccidia-free SPF chickens. Positive serum against *E. tenella* was collected from SFP chickens artificially infected with *E. tenella*. Polyclonal antibodies against Et-rROPK-Eten5-A and rEt-GRA12 were produced as previously described [27]. Briefly, 100 μg rEt-ROPK-Eten5-A or rEt-GRA12 was emulsified with Complete Freund’s adjuvant, then injected subcutaneously into female BALB/c mice (4–6 weeks old), followed by two boosters at the same dose. The titers of polyclonal antibodies were examined using ELISA for rEt-ROPK-Eten5-A or rEt-GRA12 as the antigen.

### 2.6. Immunoblotting and Immunofluorescence Assay

Recombinant Et-ROPK-Eten5-A, Et-GRA12, Et-SAG13 and Et-SAG were analyzed using Western blot assays with anti-*E. tenella* chicken sera. The recombinant proteins were separated in 12% SDS-PAGE and transferred to nitrocellulose membranes (Millipore, Bedford, MA, USA). Membranes were treated with 5% skim milk and incubated with chicken serum against *E. tenella* (1:500 dilutions) overnight at 4 °C. The membrane was incubated with horseradish peroxidase (HRP)-conjugated goat anti-chicken IgY (Sigma, Saint Louis, MO, USA)) for 1 h at 37 °C, and proteins were visualized using chemiluminescence reagents (CoWin Biotech Co., Ltd., Beijing, China) and film exposures. Coccidia-free SPF chicken serum was used as a control. Sporozoite excystation and purification were consistent with a previous study [28]. The merozoites were purified from infected chicken ceca, as published previously [29]. For immunofluorescence, the purified sporozoites and merozoites were adhered to coverslips precoated with poly-lysine and fixed with 4% (*w/v*) paraformaldehyde. The coverslips were washed, and the parasites were permeabilized with 0.1% Triton and incubated with mouse anti-Et-ROPK-Eten5-A serum (1:200) for 1 h at 37 °C. FITC- or Cy3-conjugated antibodies were used for labeling (Sigma, Saint Louis, MO, USA). DNA was stained with Hoechst 33,258 (Sigma, Saint Louis, MO, USA). Images were obtained using a Leica confocal microscope system (Leica, TCS SP52, Heidelberg, Germany).

### 2.7. The Protective Efficacy Comparison of Four Recombinant Sporozoite Proteins

**Figure 1:**
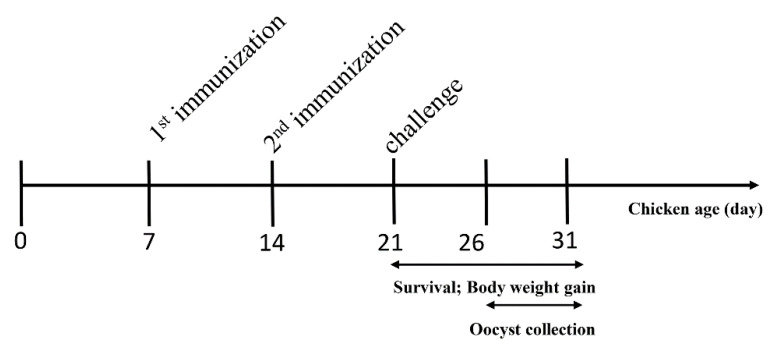
Schematic outline of the experimental design.

Fourteen-day-old chickens were weighed and randomly divided into six groups of 10 chickens per group. Animal experiments were performed as shown in Figure 1. Experimental groups were intramuscularly vaccinated with 100 µg corresponding recombinant protein per chicken in the thigh. The challenged control group and unchallenged control chickens were injected with Freund’s adjuvant. One week after primary immunization, a booster immunization was administered at the same dose. Seven days after the booster vaccination, all chickens were infected orally with 10,000 freshly sporulated *E. tenella* oocysts, and the unchallenged group received phosphate buffer saline (PBS). The protective efficacy was evaluated based on the survival rate (%), lesion score, body weight gain, relative body weight gain (%), oocyst output and oocyst decrease rate (%). Cecum lesion score was observed on the sixth day post-challenge and recorded consistent with a previous description [30]. Oocyst output was counted using a McMaster egg-counting chamber after each challenge infection. Total feces of each group was collected, mixed and weighed within five days. Three samples were randomly selected to calculate the oocysts per gram (OPG) per sample. The average amount of oocyst output per chicken was calculated. Decreases in oocyst rates were calculated as (oocyst output from positive control chickens—oocyst output from vaccinated chickens) × 100/oocyst output from positive control chickens. Body weight gain of chickens was calculated as weight at the time of slaughter—weight at the time of challenge. The relative body weight gain rate was calculated as weight gain of the experimental group × 100/weight gain of the unchallenged group.

### 2.8. Statistical Analysis

Graphs were created, and statistical analyses were performed, using Graph Pad Prism (San Diego, CA, USA). Graphs represent means, and error bars represent standard errors of means. All data were analyzed using *t*-tests. *p*-values are represented by asterisks in figures as follows: * *p* < 0.05, ** *p* < 0.01, and *** *p* < 0.001. All *p* < 0.05 were considered significant.

## 3. Results

### 3.1. Screening and Characterization of Four Sporozoite Antigens

**Figure 2:**
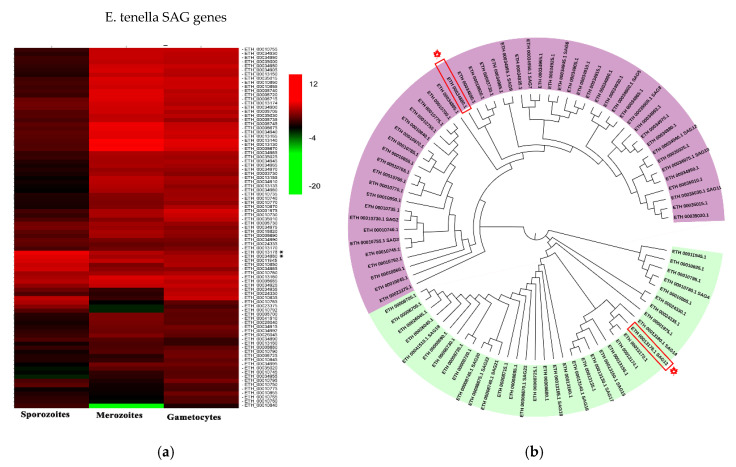
Expression profile and evolutionary analysis of *E. tenella* SAG proteins. (**a**) Transcription levels of surface antigen (SAG) genes in sporozoites, merozoites and gametocytes were obtained from ToxoDB. The heatmap of the expression profile is drawn. Et-SAG and Et-SAG13 are indicated with asterisks (*). (**b**) The amino acid sequences were downloaded from ToxoDB. A phylogenetic tree was constructed using the neighbor-joining method in MEGA software (version 5.05). Et-SAG and Et-SAG13 are indicated with asterisks (*). The light purple and light green represent different branches of the SAG family.

Surface antigens and secreted proteins are considered vaccine candidates because the sporozoite stage is a critical stage for invasion. Therefore, screening for surface antigens or secreted antigens that are highly expressed at the sporozoite stage is significant for the development of anticoccidial vaccines. The present study analyzed the expression patterns of the SAG family proteins in different stages of *E. tenella* (Figure 2a) [26]. Based on the expression patterns, we selected two highly expressed SAG antigens at the sporozoite stage (ETH_00013178 and ETH_00034880). ETH_00013178 and ETH_00034880 were identified in a previous study and named Et-SAG13 and Et-SAG [31]. According to the homology analysis of the phylogenetic tree, Et-SAG13 and Et-SAG belonged to different branches (Figure 2b).

**Figure 3:**
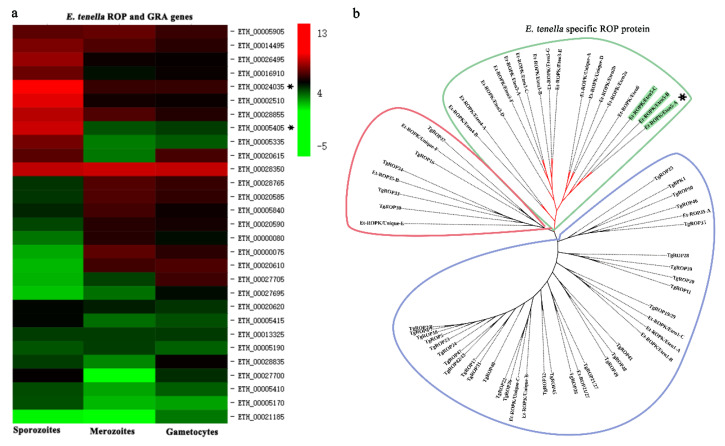
Expression profile and evolutionary analysis of *E. tenella* ROP proteins. (**a**) Transcription levels of ROP genes in sporozoites, merozoites and gametocytes were obtained from ToxoDB. The heatmap of the expression profile is drawn. Et-ROPK-Eten5-A and Et-GRA 12 are indicated with asterisks (*). (**b**) A phylogenetic tree of *E. tenella* and *T. gondii* ROP proteins was constructed using the neighbor-joining method in MEGA software (version 5.05). Et-ROPK-Eten5-A is indicated with an asterisk. The Et-ROPK-Eten5 subfamily is indicated with asterisks (*) and green background. *E. tenella* specific proteins are circled in green. ROP proteins from different branches are circled in red and blue, respectively.

**Figure 4:**
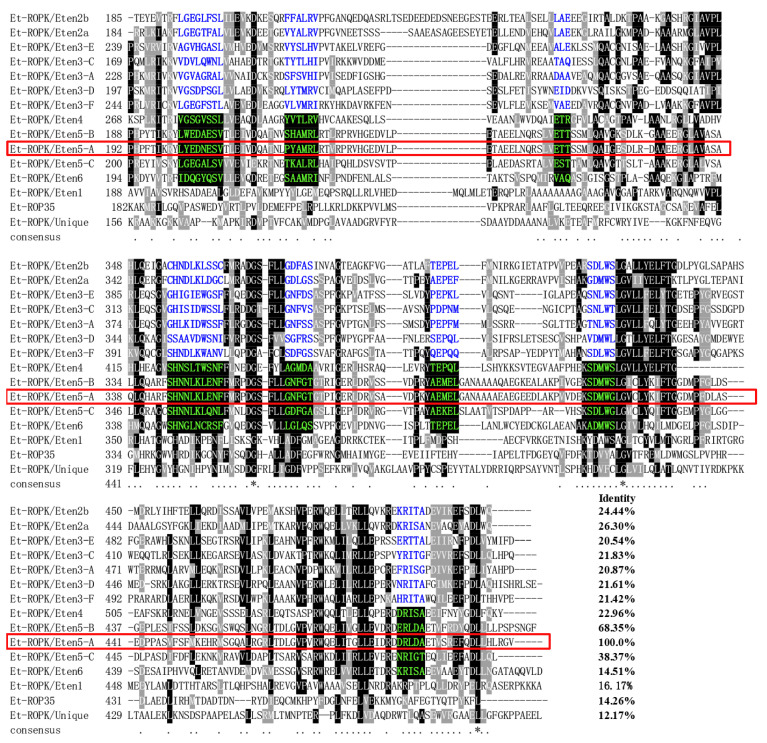
Sequence alignment and conserved motif analysis of *E. tenella* rhoptries (ROP) proteins. Multiple sequence alignment was performed using Clustal X software version 1.83. The regions of high identity and similarity between ROP sequences are shown as black and gray columns, respectively. The identity of Et-ROPK-Eten5-A with each ROP is shown at the end of the alignment. The conserved motif regions of the kinase domain are highlighted. The conserved motifs of predicted noncanonical catalytic mechanisms are highlighted with blue letters. The conserved motifs of likely inactive rhoptry kinase subfamilies are highlighted with a black background and green letters. The catalytic activities of the conserved motifs were predicted in a previous report [32]. TheEt-ROPK-Eten5-A in this study is circled in red frame.

Similarly, the expression patterns of ROP family and GAR family proteins in different stages of *E. tenella* were compared [26]. An ROP protein and a GRA protein (ETH_00005405 and ETH_00024035) were highly expressed at the sporozoite stage and selected (Figure 3a). Sequence alignment revealed that ETH_00024035 showed homology with GRA12 from *Toxoplasma gondii*, which was named Et-GRA12. A previous study revealed that ETH_00005405 was a specific ROP protein of *E. tenella* and belonged to the ROPK-Eten5 subfamily [32], named Et-ROPK-Eten5-A. Phylogenetic tree analysis showed that the Et-ROPK-Eten5 subfamily was a cluster alone, and Et-ROPK-Eten5-A belonged to this cluster (Figure 3b). Multiple sequence alignment revealed that Et-ROPK-Eten5-A shared high similarity with the Et-ROPK-Eten5-B (68.35%), but only 12.17%–26.30% with other clusters of ROP proteins (Figure 4). Comparison of the active site residues showed that Et-ROPK-Eten5-A contained a classical protein kinase domain at 265 to 499 amino acids and eight conserved motifs, including LYEDNESV, PYAMRL, ETT, SHNNLKLENF, GNFGT, AEMEL, SDMWG and DRLDA. However, Et-ROPK-Eten5-A lacked critical aspartates that participated in the kinase catalytic activity [32].

### 3.2. Cloning and Expression of the Four Sporozoite Proteins

**Figure 5:**
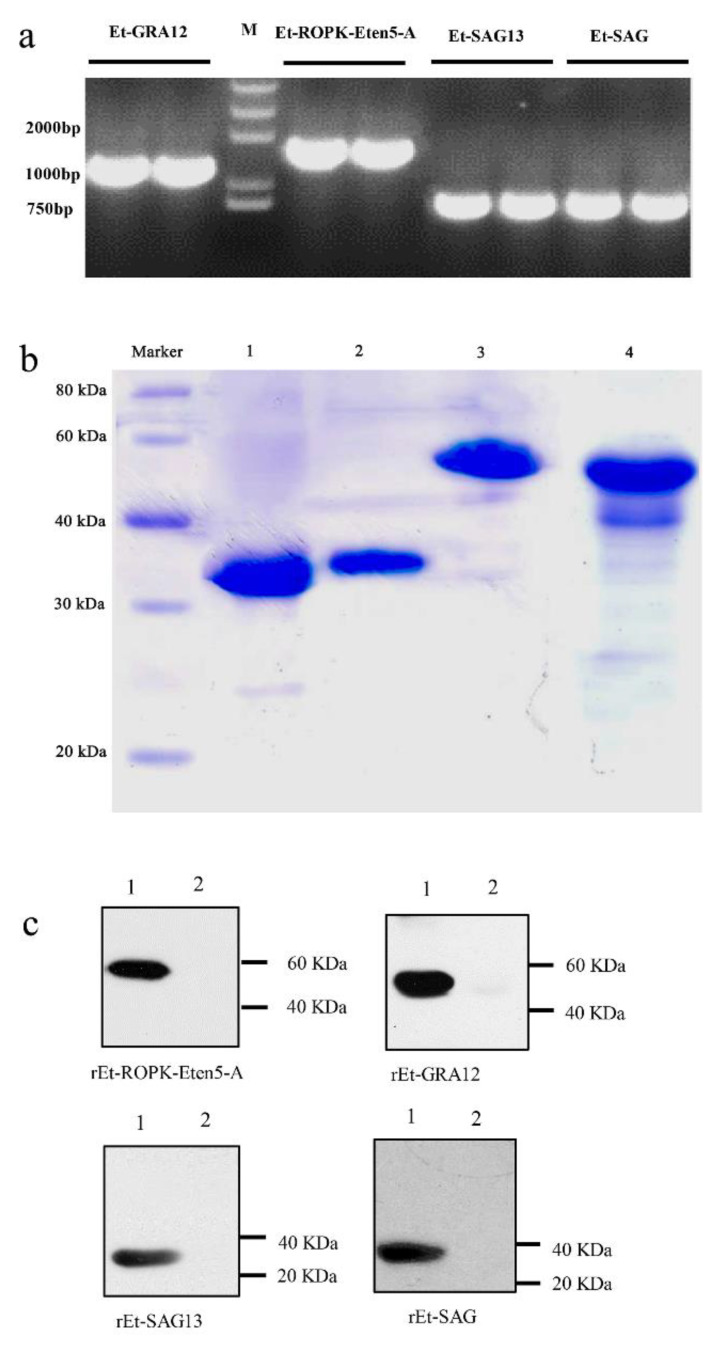
Cloning, expression and immunogenicity identification of four sporozoite recombinant proteins. (**a**) The full coding sequences of Et-ROPK-Eten5-A, Et-GRA12, Et-SAG and Et-SAG13 without the signal peptide sequences were amplified from cDNA. (**b**) Purification of recombinant proteins. Lane 1, purified rEt-SAG13 protein; Lane 2, purified rEt-SAG protein; Lane 3, purified rEt-ROPK-Eten5-A protein; Lane 4, purified rEt-GRA12 protein. (**c**) Western blotting analysis of recombinant proteins. Lane 1, *E. tenella* positive serum; Lane 2, negative serum.

The full-length coding sequences of Et-ROPK-Eten5-A, Et-GRA12, Et-SAG13 and Et-SAG were 1524, 1221, 789 and 762 bp nucleotides, respectively, which individually encoded 508, 407, 263 and 254 amino acids with predicted molecular weights of ~56, ~45, ~29 and ~29 kDa, respectively (Figure 5a). There was a signal peptide cleavage site in the Et-ROPK-Eten5-A and Et-GRA12 sequences, which indicated that both proteins were secretory proteins. The predicted molecular weights of Et-ROPK-Eten5-A and Et-GRA12 without the signal peptide were ~51 kDa and ~41 kDa, respectively. The expression of rEt-ROPK-Eten5-A, rEt-GRA12, rEt-SAG13 and rEt-SAG proteins in *Escherichia coli* transformed with the pET28a (+) plasmid were confirmed using SDS-PAGE and were consistent with the predicted sizes of ~57, ~47, ~35 and ~35 kDa, including a His-tag, respectively (Figure 5b).

### 3.3. Immunoblot Analysis of the Four Recombinant Sporozoite Proteins

The rEt-ROPK-Eten5-A, rEt-GRA12, rEt-SAG13 and rEt-SAG proteins were tested using chicken *E. tenella*-positive serum, and a coccidia-free chicken serum was used as a control. The results showed that all four recombinant proteins reacted with positive serum, but no band was observed in the negative control (Figure 5c).

### 3.4. Immunolocalizations of Endogenous Et-ROPK-Eten5-A and Et-GRA12

**Figure 6:**
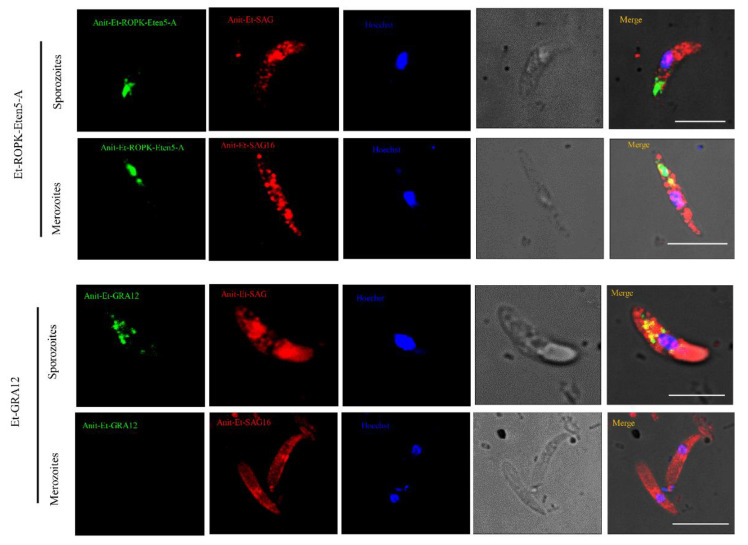
Immunofluorescence localization of Et-ROPK-Eten5-A and Et-GRA12 in different stages of *E. tenella*. Immunofluorescence localization of Et-ROPK-Eten5-A and Et-GRA12 in sporozoites and merozoites using mouse anti-rEt-ROPK-Eten5-A and mouse anti-r Et-GRA12 serum. Scale bars: 10 μm. Et-SAG and Et-SAG16 (red) served as parasite surface markers. The morphology of parasites under a light microscopes is shown in the penultimate column.

Because Et-ROPK-Eten5-A and Et-GRA12 were not identified previously, localization studies were performed. The distributions of Et-ROPK-Eten5-A and Et-GRA12 in sporozoites and merozoites stages of *E. tenella* were assessed using immunofluorescence and a mouse anti-rEt-ROPK-Eten5-A antibody and mouse anti-Et-GRA12 antibody. Et-ROPK-Eten5-A was distributed in the apical area of sporozoites (Figure 6), and it distributed in the apical pole of merozoites as a slender form. Et-GRA12 was scattered in granular form in sporozoites (Figure 6), and it was not detected in the body of merozoites.

### 3.5. Recombinant Et-ROPK-Eten5-A Induces Effective Protection against E. tenella

**Figure 7:**
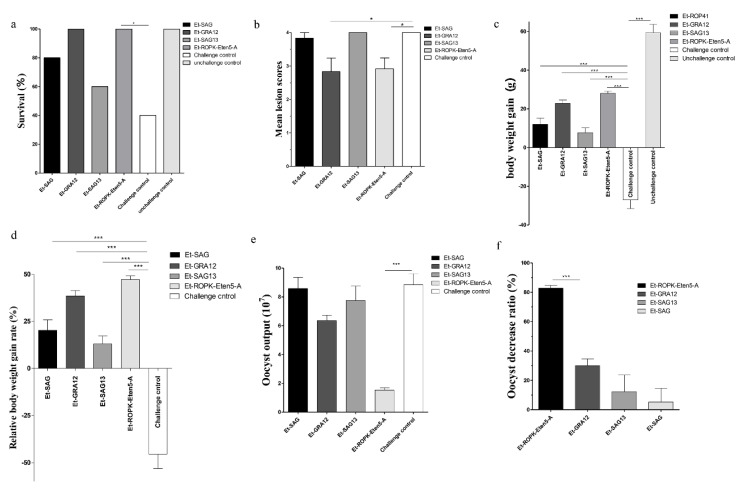
Protective efficacy of the four sporozoite antigens against *E. tenella.* (**a**) Effects of vaccination with four sporozoite antigens on survival rate, (**b**) cecum lesion score, (**c**) body weight gain, (**d**) relative body weight gain, (**e**) oocyst output and (**f**) oocyst decrease rate. Data are the means ± SD (error bars) of three independent experiments. All data were analyzed using *t*-tests. *p*-values are represented by asterisks as follows: * *p* < 0.05, and *** *p* < 0.001.

To evaluate the protective efficacy of rEt-ROPK-Eten5-A, rEt-GRA12, rEt-SAG13 and rEt-SAG proteins, groups of chickens were challenged with *E. tenella*. The survival rate was significantly improved in rEt-ROPK-Eten5-A- and rEt-GRA12-immunized chickens compared to the challenged control group (Figure 7a). There was a significant reduction in the mean lesion score of the rEt-ROPK-Eten5-A (*p* < 0.05) and rEt-GRA12 (*p* < 0.05) immunized chickens compared to unimmunized chickens (Figure 7b). No significant difference in rEt-SAG13 (*p* > 0.05) or rEt-SAG (*p* > 0.05) immunized birds was observed compared to unimmunized chickens. The average body weight gains of rEt-ROPK-Eten5-A (28.00 ± 3.50 g, *p* < 0.001), rEt-GRA12 (22.80 ± 5.53 g, *p* < 0.001), rEt-SAG13 (7.70 ± 7.80 g, *p* < 0.001) and rEt-SAG (12.00 ± 10.39, *p* < 0.001) immunized chickens were significantly higher than those of adjuvant-immunized chickens (−27 ± 14.24 g) and corresponded to relative body weight gain rates of 47% (rEt-ROPK-Eten5-A, *p* < 0.001), 38% (rEt-GRA12, *p* < 0.001), 12% (rEt-SAG13, *p* < 0.001) and 20% (rEt-SAG, *p* < 0.001) compared to control immunized chickens (−46%), respectively (Figure 7c,d). The decreases in oocyst output were 82.75% in the rEt-ROPK-Eten5-A-immunized group and 29.95%, 12.29% and 5.18% in the rEt-GRA12-, rEt-SAG13- and rEt-SAG-immunized groups compared to unimmunized challenged chickens (Figure 7e,f). These results demonstrated that the four recombinant sporozoite proteins triggered a heterologous immunoprotective effect in *E. tenella*-infected chickens, and the rEt-ROPK-Eten5-A protein had the best protection efficiency.

## 4. Discussion

*E. tenella* belongs to the phylum Apicomplexa, and it has unique secretory organelles (micronemes, rhoptries and dense granules) that secrete proteins for the invasion process [12,33], which are considered vaccine candidates [9]. The present study focused on highly expressed proteins in sporozoites, including two secreted antigens and two surface antigens. *E. tenella* SAG genes are classified into four categories, each on a different chromosome. Family A is common to all species, but family B is restricted to *E. tenella* and *E. necatrix* [31]. Et-SAG13 belongs to family B, and Et-SAG belongs to family A. Phylogenetic tree analysis based on homology revealed that the SAG family may be divided into two clades, and the Et-SAG13 and Et-SAG proteins belonged to different branches. A previous study identified three ROPK subclades of particular interest, including a structurally conserved N-terminal extension to the kinase domain (NTE), *E. tenella*-specific expansion and a basal cluster [32]. Phylogenetic tree analysis showed that Et-ROPK-Eten5-A belonged to the Et-ROPK-Eten5 subfamily, which is the ROPK subclade of the *E. tenella*-specific expansion cluster. Our sequence alignment showed that Et-ROPK-Eten5-A contained a classical protein kinase domain and eight conserved motifs (LYEDNESV, PYAMRL, ETT, SHNNLKLENF, GNFGT, AEMEL, SDMWG and DRLDA) of the inactive rhoptry kinase subfamilies. However, Et-ROPK-Eten5-A lacked critical aspartates that participated in the kinase catalytic activity, and it is considered a pseudokinase [32].

Because Et-ROPK-Eten5-A and Et-GRA12 were not identified previously, localization studies were performed. The distribution of Et-ROPK-Eten5-A and Et-GRA12 in sporozoites and merozoites of *E. tenella* was assessed using immunofluorescence and a mouse anti-rEt-ROPK-Eten5-A antibody and mouse anti-Et-GRA12 antibody. Et-ROPK-Eten5-A was distributed in the apical area of sporozoites, which was similar to the localization of Et-ROP1 protein [34]. A previous report showed that free second-generation merozoites had a long, slender body with two rhoptries at the apical pole [35]. Et-ROPK-Eten5-A was distributed in the apical pole of merozoites with a slender form in our study, which is consistent with the distribution of identified ROP proteins. Et-GRA12 was scattered in a granular form in sporozoites, which is similar to the localization of dense granular proteins in the tachyzoites of *Neospora caninum* [36].

*E. tenella* is one of the most prevalent and pathogenic species of *Eimeria* infecting chickens [4]. *E. tenella* infection may result in severe lesions of the ceca, body weight loss, hemorrhagic diarrhea, hemorrhage and death [4,37]. Anticoccidial drugs are predominantly used to control *Eimeria* infection. However, the rise in drug resistance and public pressure for restrictions on foodborne animal chemicals continue to drive the development of anti-coccidiosis vaccines [3,38,39]. Therefore, it is urgent to develop safe and effective vaccines against avian coccidiosis [7,21,40]. The sporozoite stage plays a critical role in invasion. Therefore, the screening of sporozoites that highly expressed secreted antigens or surface antigens is crucial for the development of anticoccidial vaccines. The strategy for these vaccines is to block parasite infection by disturbing the invasion process. The present study successfully obtained recombinant Et-ROPK-Eten5-A, Et-GRA12, Et-SAG13 and Et-SAG proteins and assessed their immune protective efficiency against *E. tenella*. We detected the reaction of four recombinant proteins with positive serum of *E. tenella* infected chickens by Western blotting, respectively. The results implied that the chicken immune system would produce antibodies against these proteins under natural infection. Our results showed that vaccination with rEt-ROPK-Eten5-A significantly increased the survival rate and body weight gains, decreased the oocyst output and alleviated cecum lesions compared to the challenge control group. All of these results suggest that rEt-ROPK-Eten5-A provided effective protection for chickens against *E. tenella*. However, the protective immunities of rEt-GRA12, rEt-SAG13 and rEt-SAG proteins were not satisfactory compared to rEt-ROPK-Eten5-A. The groups immunized with rEt-GRA12 protein showed significantly increased body weight and reduced cecum lesion scores. However, vaccination with rEt-GRA12 was not effective in oocyst shedding, which suggests that rEt-GRA12 may not be a good vaccine candidate for controlling the oocyst output in infected chickens. For the rEt-SAG13 and rEt-SAG proteins, the index of survival rate, oocyst output reduction and weight gain were not as good as rEt-ROPK-Eten5-A, which suggest that they are not ideal antigens for vaccine.

A previous study investigated several sporozoite proteins of *Eimeria* as anticoccidial vaccine candidates. The efficacy of a recombinant sporozoite antigen (EtMIC1) protein in protecting against a homologous challenge was evaluated and only showed partial protection against homologous challenge in chickens [17]. The sporozoite-specific SAG1 also induced partial protective immunity as a subunit vaccine [19,20,21]. Antigens SO7 and ETRHO1 had been investigated to locate in the sporozoite refractile bodies and induce both cellular and humoral immune responses in immunized chickens. The oocyst reduction rate of rEt-ROPK-Eten5-A (82.75%) is better than that of SO7 (74.46%) [16,18]. The relative weight gain of the chickens immunized with Et-ROPK-Eten5-A was only 47%, but the weight gains of chickens immunized with SO7 and ETRHO1 were 86.77% and 87%, respectively. This seems to indicate that Et-ROPK-Eten5-A has less protective effect on intestinal injury in chickens than SO7 and ETRHO1 as previously reported. However, it is worth noting that different *E. tenella* strains have different levels of virulence; our challenge dose resulted in a weight loss of 46% in the unimmunized control group and a mortality rate of 60%, whereas the previous study using SO7 and ETRHO1 proteins reported weight gain rates of 69.79% and 47.80% in the respective unimmunized control groups. Therefore, rEt-ROPK-Eten5-A is considered an effective anticoccidial vaccine. The combination of rEt-ROPK-Eten5-A and other effective proteins (such as SO7 and ETRHO1) may be a new direction for the development of anticoccidial vaccines in the future. IMP1 was identified as an anticoccidial vaccine candidate, and it is localized in the sporozoite cell membrane [22]. However, the use of IMP1 in combination with a powerful immunological adjuvant (CD40L) did not improve the index of weight gain rate and oocyst decrease rate more than rEt-ROPK-Eten5-A [23].

## 5. Conclusions

In summary, we identified and characterized four sporozoite proteins from *E. tenella*, including two secreted antigens and two surface antigens. We presented the locations of Et-ROPK-Eten5-A and Et-GRA12 and provided novel insights into the biological functions of these proteins. Our study demonstrated that vaccination with rEt-ROPK-Eten5-A significantly increased the survival rate and body weight gains, decreased the oocyst output and alleviated cecum lesions, which indicate that Et-ROPK-Eten5-A may be an effective candidate for the development of vaccines against *E. tenella*.

## Supplementary Materials

The following are available online at https://www.mdpi.com/2076-393X/8/3/452/s1, Table S1: Primers used in this study title.
