# Supplementary material for: A Novel Rhoptry Protein as Candidate Vaccine against Eimeria tenella Infection
Source: Vaccines (Basel). 2020 Aug 12;8(3):452. doi: 10.3390/vaccines8030452 (PMC7565193; doi:10.3390/vaccines8030452)
Supplement: Supplementary file 1 [file vaccines-08-00452-s001.pdf]

vaccines

MDPI

Table S1 Primers used for this study.

Primer name		Sequence
Et- ROP41	F	5′- AGCAAATGGGTCGCGGATCCGAACCTCCCCGAGTCAACCT -3′
	R	5′- TCGAGTGCGGCCGCAAGCTTATCCTGGAACTCCCTGGACACC -3′
Et- GRA12	F	5′- AGCAAATGGGTCGCGGATCCGTTGGCGTTTACTCTCCTGA -3′
	R	5′- TCGAGTGCGGCCGCAAGCTTATTTCTCTTCGAGGCCGAAT -3′
Et-SAG	F	5′- AGCAAATGGGTCGCGGATCCATGGCGCCTCTTACACTACT -3′
	R	5′- TCGAGTGCGGCCGCAAGCTTTACTGCAGCCATCCCAAGCG -3′
Et-SAG13	F	5′- AGCAAATGGGTCGCGGATCCATGTCTCGACTTGGCCTGTT -3′
	R	5′- TCGAGTGCGGCCGCAAGCTTACTCAACAGAATTAACGCGA -3′
pET28a	F	5′- GGATCCGCGACCCATTTGCT -3′
	R	5′- AAGCTTGCGGCCGCACTCGA -3′
